# Stimuli-responsive nucleic acid-functionalized metal–organic framework nanoparticles using pH- and metal-ion-dependent DNAzymes as locks†
†Electronic supplementary information (ESI) available. See DOI: 10.1039/c7sc01765k



**DOI:** 10.1039/c7sc01765k

**Published:** 2017-06-06

**Authors:** Wei-Hai Chen, Xu Yu, Alessandro Cecconello, Yang Sung Sohn, Rachel Nechushtai, Itamar Willner

**Affiliations:** a Institute of Chemistry , Center for Nanoscience and Nanotechnology , The Hebrew University of Jerusalem , Jerusalem 91904 , Israel . Email: willnea@vms.huji.ac.il ; Fax: +972-2-6527715 ; Tel: +972-2-6585272; b Institute of Life Science , The Hebrew University of Jerusalem , Jerusalem 91904 , Israel

## Abstract

A versatile approach to modify metal–organic framework nanoparticles (NMOFs) with nucleic acid tethers, using the “click chemistry” method is introduced. The nucleic acid-functionalized NMOFs are used to prepare stimuli-responsive carriers of loads (fluorescence probes or anti-cancer drugs). Two different stimuli-responsive nucleic acid-based NMOFs are presented. One system involves the preparation of pH-responsive NMOFs. The NMOFs are loaded with fluorophores or doxorubicin anti-cancer drug and locked in the NMOFs by pH-responsive DNA duplex capping units. At pH = 5.0 the capping units are unlocked, leading to the release of the loads. The AS1411 aptamer is conjugated to the locking units as the targeting unit for the nucleolin biomarker present in cancer cells. The pH-responsive doxorubicin-loaded NMOFs and, in particular, the AS1411 aptamer-modified pH-responsive NMOFs reveal selective, targeted, cytotoxicity toward MDA-MB-231 breast cancer cells. A second system involves the synthesis of NMOFs that are loaded with fluorophores or doxorubicin and capped with metal-ion-dependent DNAzyme/substrate complexes as locking units (metal ion = Mg^2+^ or Pb^2+^ ions). In the presence of the respective metal ions, the nucleic acid locking units are cleaved off, resulting in the release of the loads. Also, “smart” Mg^2+^-ion-dependent DNAzyme capped doxorubicin-loaded NMOFs are synthesized *via* the integration of the ATP aptamer sequence in the loop domain of the Mg^2+^-dependent DNAzyme. The unlocking of these NMOFs proceeds effectively only in the presence of ATP and Mg^2+^ ions, acting as cooperative triggers. As ATP is over-expressed in cancer cells, the “smart” carrier provides sense-and-treat functions. The “smart” ATP/Mg^2+^-triggered doxorubicin-loaded NMOFs reveal selective cytotoxicity toward MDA-MB-231 cancer cells. Beyond the use of the metal-ion-dependent DNAzymes as ion-responsive locks of drug-loaded NMOF carriers, the DNAzyme-capped fluorophore-loaded NMOFs are successfully applied as functional units for multiplexed ion-sensing and for the design of logic-gate systems.

## Abstract 2

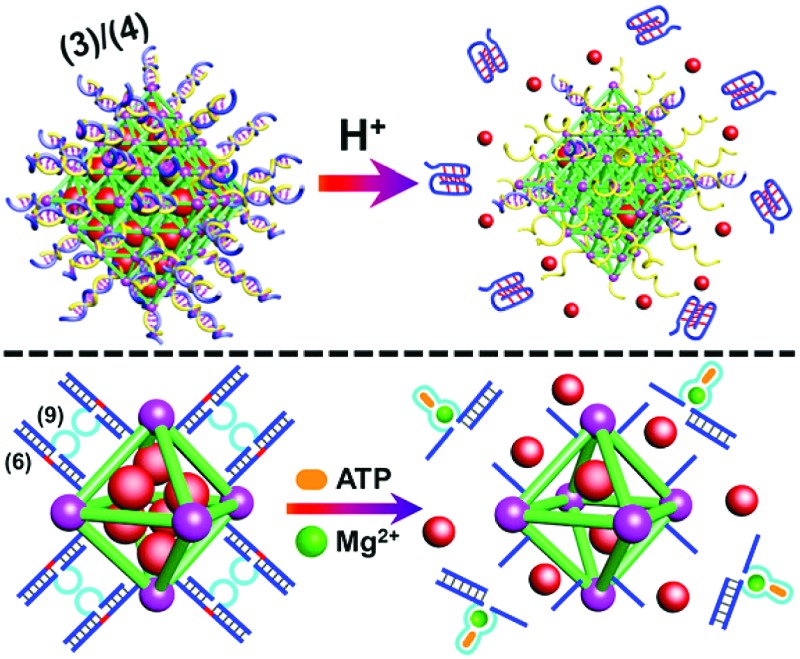
Drug-loaded DNA-capped metal–organic framework nanoparticles are unlocked by pH or Mg^2+^ ions/ATP triggers, resulting in the release of the loads.

## Introduction

Metal–organic frameworks (MOFs) represent a broad class of organic/inorganic porous materials that have attracted substantial research efforts in recent years.^1^ Different applications of the porous MOFs as functional carriers have been demonstrated, and these include their use as carriers for catalysts,^2^ matrices for the storage of gases,^3^ frameworks for carrying drugs and controlled delivery systems,^4^ materials for sensing,^5^ and functional materials for improving fuel cells performance.^6^

The base sequence of nucleic acids encodes substantial structural and functional information into the biopolymer.^7^ This includes the signal-triggered reconfiguration of nucleic acids such as the pH-induced reconfiguration of cytosine-rich nucleic acids into i-motif structures,^8^ or the assembly of guanosine-rich sequences into G-quadruplexes, in the presence of ions, *e.g.* K^+^, Pb^2+^, or Sr^2+^ ions.^9,10^ Similarly, sequence-specific nucleic acids reveal catalytic properties, and metal-ion-dependent catalytic nucleic acids (M^*n*+^ = Mg^2+^, Pb^2+^, Hg^2+^, Cd^2+^ and more) have been developed.^11^ These unique functions of nucleic acids were applied for the development of stimuli-responsive drug carriers. Mesoporous SiO_2_ nanoparticles,^12^ hydrogels,^13^ microcapsules,^14^ or liposomes^15^ were loaded with drugs (or drug models) and locked by stimuli-responsive nucleic acid capping units. In the presence of appropriate triggers, such as pH,^16^ enzymes,^17^ aptamer/ligand complexes,^18^ or metal-ion-dependent DNAzymes,^19^ the nano/micro carriers were unlocked, resulting in the release of the loads.

The high porosity of MOFs, the ability to modify the MOFs using surface functionalities, and particularly, the feasibility of synthesizing nano-sized metal–organic framework nanoparticles (NMOFs), turn these nanomaterials into ideal drug-carrying matrices. Although several studies reported on the synthesis of stimuli-responsive MOFs using chemical capping functionalities,^20^ the preparation of stimuli-responsive DNA/MOF hybrid systems is scarce and relatively unexplored. Only recently, we reported on the synthesis of substrate-loaded microcrystalline MOFs capped by pH-responsive or K^+^ ion-stabilized G-quadruplex capping units.^21^ The pH- or crown-ether-induced unlocking of these MOFs was demonstrated. Nonetheless, these microcrystalline MOFs cannot permeate into cells, and their use as drug-delivery systems is limited. Accordingly, the development of stimuli-responsive nucleic acid-functionalized nano-sized metal–organic frameworks, NMOFs, is needed.

In the present study we report on the synthesis and loading of nucleic acid-functionalized, stimuli-responsive NMOFs. The nucleic acid capping units are composed of pH-responsive nucleic acids or a metal-ion-dependent DNAzyme unit. The loads trapped in the NMOFs are fluorescent dyes (acting as drug analoges) or the doxorubicin anti-cancer drug. In the presence of appropriate triggers, pH or metal ions, the NMOFs are unlocked, leading to the release of the loads. It should be noted that the previously reported nucleic acid capping units,^21^ used to lock the microcrystalline MOFs, could not be adapted to cap the loaded NMOFs (leakage of the loads was observed in the absence of an external trigger). This forced us to develop a new strategy to synthesize the stimuli-responsive, gated, DNA/NMOF systems. This involves the covalent modification of the NMOF with nucleic acid tethers using the “click-chemistry” principle. We further demonstrate the incorporation of the NMOFs into cancer cells and study the cytotoxicity of doxorubicin-loaded NMOFs towards cancer cells. We also discuss other applications of the stimuli-responsive NMOFs as sensors, sense-and-treat systems, and functional components for logic-gate operations. It should be noted that the related nucleic acid-based locked mesoporous SiO_2_ nanoparticles as drug carriers were previously reported.^12^ However, the NMOFs reveal the following advantages over the SiO_2_ nanoparticles: (i) the ordered repeat unit cell ions/ligands in NMOFs allow the precise modification of the ligand with the nucleic acid, and (ii) nucleic acid-modified mesoporous SiO_2_ nanoparticles tend to aggregate and precipitate, while the nucleic acid-functionalized NMOFs are stable as suspendable mixtures. This turns the NMOFs into superior drug carriers for medical applications.

## Results and discussion

**Fig. 1:**
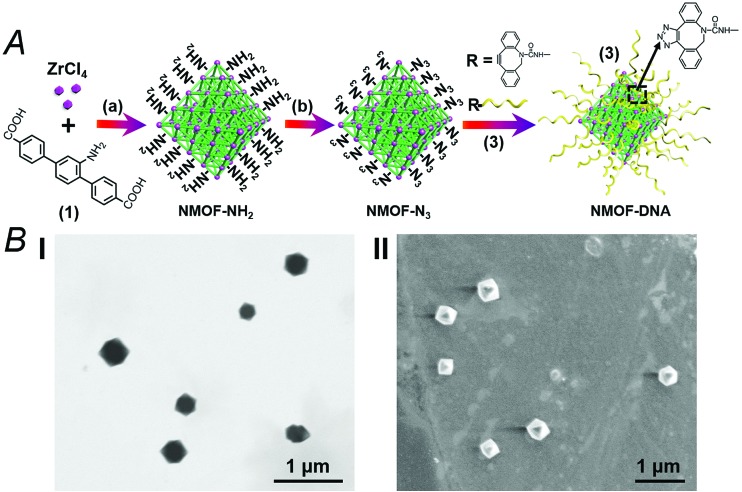
(A) Synthesis of the nucleic acid-functionalized UiO-68 NMOF particles. (a) DMF, 80 °C, 5 days. (b) *t*BuONO and TMSN_3_, THF, overnight. (B) A TEM image, panel I, and SEM image, panel II, of the UiO-68 NMOFs.

**Fig. 2:**
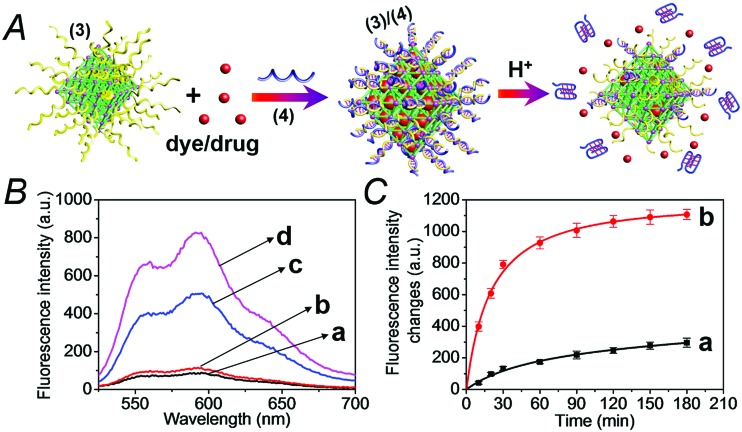
(A) The preparation of the dye/drug-loaded NMOFs capped and locked by the (**3**)/(**4**) duplex and the pH-induced unlocking of the NMOFs *via* the dissociation of the duplex locks through the release of i-motif structures. The unlocking of the capping units leads to the release of the dye/drug loads. (B) The fluorescence spectra of the released doxorubicin (DOX) from the NMOFs: (a) at pH = 7.0, after 30 minutes, (b) at pH = 7.0, after 60 minutes, (c) at pH = 5.0, after 30 minutes, and (d) at pH = 5.0, after 60 minutes. (C) Time-dependent fluorescence changes upon treatment of the DOX-loaded NMOFs at: (a) pH = 7.4, (b) pH = 5.0. The fluorescence corresponds to the released DOX.

The synthesis of the DNA-gated metal–organic framework nanoparticles (NMOFs) is depicted in Fig. 1(A). Amine-functionalized triphenyl carboxylic acid (**1**) was reacted with ZrCl_4_ to yield the porous NMOFs.^22^ The size of the porous nanoparticles was in the range of 250–300 nm. The NMOFs had a bipyramidal structure, as shown in Fig. 1(B). BET measurements indicated a surface area of *ca.* 1160 m^2^ g^–1^ and pore size corresponding to *ca.* 1.58 nm. The amine functionalities associated with the NMOFs were transformed to azide functionalities in the presence of *t*-butyl nitrite and trimethylsilyl azide and modified with a nucleic acid using the click chemistry principle. The amine-modified nucleic acid (**2**) was reacted with dibenzocyclooctyne-sulfo-*N*-hydroxysuccinimidyl ester (DBCO-sulfo-NHS ester), and the resulting DBCO-modified nucleic acid (**3**) was conjugated to the azide groups linked to the NMOFs. Fig. 2(A) depicts the loading of the NMOFs with the dye/drug loads and the pH-induced unlocking and release of the loads. The (**3**)-functionalized NMOFs were loaded with the respective dye/drug and capped by the hybridization with the nucleic acid (**4**) that is complementary to the surface-linked promoter nucleic acid (**3**). The hybrid duplex (**3**)/(**4**) is designed to include in the strand (**4**), a cytosine (C)-rich sequence that reconfigures at acidic pH into an i-motif structure. This hybrid was designed with the vision that an acidic pH exists in cancer cells, and thus, reconfiguration of (**4**) into the i-motif structure would unlock the NMOFs and release the anti-cancer drugs. Accordingly, the NMOFs were loaded with the methylene blue dye as a drug model, or with the doxorubicin anti-cancer drug. Fig. S1† depicts the time-dependent release of methylene blue from the NMOFs upon subjecting the nanoparticles to pH = 7.4 and pH = 5.0. Evidently, the release of the dye from the NMOFs is substantially more efficient at pH = 5.0. After *ca.* 180 minutes, the fluorescence intensity of the dye levels off to a saturation value, implying that the dye trapped in the NMOFs was fully released. Using an appropriate calibration curve, we estimate that *ca.* 72.2 μmol methylene blue were released per gram of NMOFs (for the evaluation of the loading of methylene blue in the NMOFs, see Fig. S2, ESI†). It should be noted that the loading of methylene blue associated with the nucleic acid-functionalized NMOFs is substantially higher (*ca.* 4-fold) compared to that of the nucleic acid-functionalized mesoporous SiO_2_ nanoparticles.^19^ The time-dependent release of the dye at pH = 7.4 also reaches a saturation value. This background release is attributed to crystalline domains with incomplete or defective capping. Fig. 2(B) depicts the fluorescence spectra of doxorubicin, DOX, released from the NMOFs at pH = 5.0 after time intervals of 30 and 60 minutes. These fluorescence spectra are compared to the fluorescence spectra of DOX released from the NMOFs at pH = 7.0. Evidently, the release of DOX from the NMOFs at pH = 7.0 is very low. Fig. 2(C) depicts the time-dependent release of DOX from the NMOFs at pH = 5.0 and pH = 7.4. From the saturated fluorescence intensity of DOX at pH = 5.0, and using an appropriate calibration curve, we estimate that *ca.* 52.8 μmol DOX were released per gram of nanoparticles (for the evaluation of the loading of DOX in the NMOFs, see Fig. S3, ESI†). The enhanced release of the different loads at pH = 5.0 is attributed to the reconfiguration of (**4**) into the i-motif structure that leads to the unlocking of the duplex capping units and the release of the dye/drug.

**Fig. 3:**
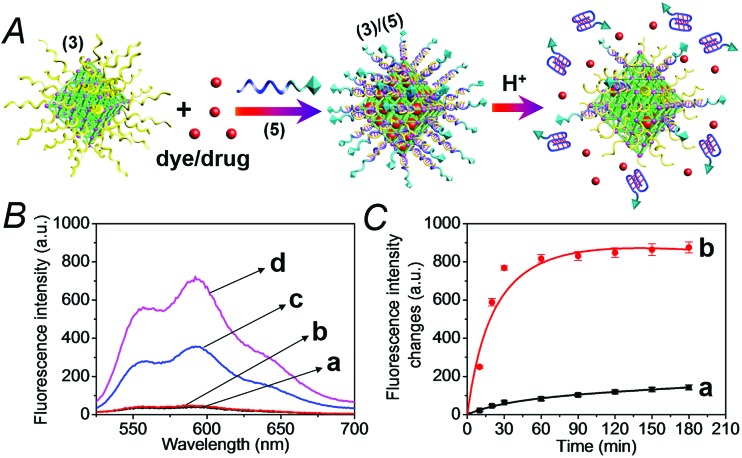
(A) Loading the (**3**)-modified NMOFs with dye/drug loads through the capping of the loaded NMOFs with duplex locks (**3**)/(**5**), where the strand (**5**) consists of the pH-responsive and AS1411 aptamer domains, and the unlocking of the NMOFs by the separation of the duplex locks through the formation of i-motif structures. (B) The fluorescence spectra of the released DOX from the NMOFs: (a) at pH = 7.0, after 30 minutes, (b) at pH = 7.0, after 60 minutes, (c) at pH = 5.0, after 30 minutes, and (d) at pH = 5.0, after 60 minutes. (C) The time-dependent fluorescence changes upon treatment of the (**3**)/(**5**)-locked DOX-loaded NMOFs at: (a) pH = 7.4 and (b) at pH = 5.0.

The doxorubicin-loaded NMOFs were further modified to include a cancer cell targeting element. Towards this end we made use of the fact that certain cancer cells include the nucleolin receptor to which the AS1411 aptamer was developed.^23^ Accordingly, the (**3**)-modified dye/doxorubicin-loaded NMOFs were hybridized with the nucleic acid (**5**), which includes in its sequence the cytosine-rich, pH-responsive domain and the AS1411 aptamer domain, shown in Fig. 3(A). Fig. S4† depicts the pH-induced release of the methylene blue dye from the NMOFs that are locked with the (**3**)/(**5**) capping units. While at pH = 7.4 inefficient release of the dye occurs, at pH = 5.0 effective release of the dye proceeds, as a result of the unlocking of the capping units through the formation of i-motif structures. Fig. 3(B) and (C) show the pH-induced release of DOX from the (**3**)/(**5**)-capped NMOFs. Fig. 3(B) shows the fluorescence spectra of the released DOX at pH = 5.0, after time intervals of 30 and 60 minutes, in comparison to the fluorescence spectra of the released DOX after the same time intervals at pH = 7.4. While the release of DOX at pH = 7.4 is negligible, effective release of DOX is observed at pH = 5.0. Fig. 3(C) shows the time-dependent release profiles of DOX from the NMOFs at pH = 5.0 and pH = 7.4.

**Fig. 4:**
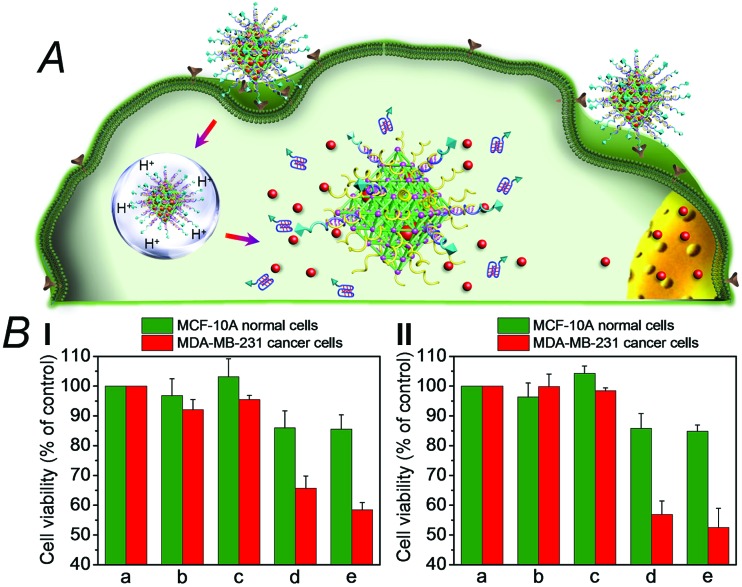
(A) Schematic targeted penetration of the (**3**)/(**5**)-locked, DOX-loaded, NMOFs into the MDA-MB-231 cancer cells and the pH-induced unlocking of the NMOFs and release of DOX in the cytoplasm. (B) Cytotoxicity of the DOX-loaded (**3**)/(**4**)- or (**3**)/(**5**)-capped NMOFs and reference systems towards MCF-10A epithelial normal breast cells (green) and MDA-MB-231 breast cancer cells (red). Panel I shows results after three days and panel II after five days. The entries correspond to (a) untreated cells, (b) cells treated with unloaded (**3**)/(**4**)-locked NMOFs, (c) cells treated with unloaded (**3**)/(**5**)-locked NMOFs, (d) cells treated with DOX-loaded (**3**)/(**4**)-locked NMOFs, and (e) cells treated with DOX-loaded (**3**)/(**5**)-locked NMOFs.

**Fig. 5:**
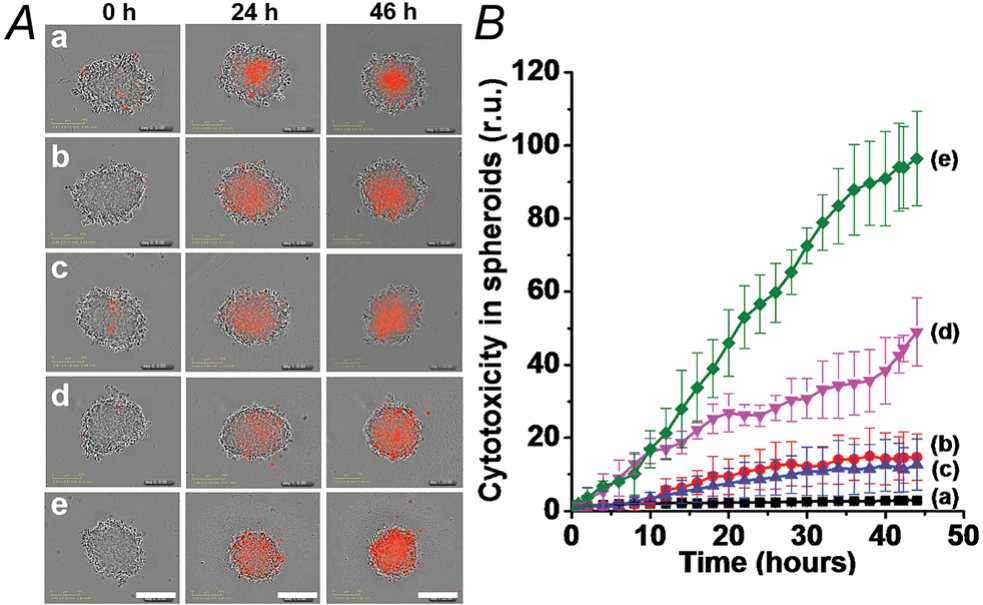
Cytotoxicity of the (**3**)/(**4**)- and (**3**)/(**5**)-capped DOX-loaded NMOFs and reference systems towards spheroid aggregates of MDA-MB-231 breast cancer cells. The cytotoxicity is evaluated by the time-dependent apoptosis of the cell aggregates, monitored colorimetrically *via* the staining of the aggregates with IncuCyte cytotoxicity reagent. (A) Typical apoptosis color responses of spheroid cell aggregates after 0 hours, 24 hours, and 46 hours. (a) Untreated cells, (b) cells treated with (**3**)/(**4**)-locked unloaded NMOFs, (c) cells treated with (**3**)/(**5**)-locked unloaded NMOFs, (d) cells treated with (**3**)/(**4**)-locked DOX-loaded NMOFs, and (e) cells treated with (**3**)/(**5**)-locked DOX-loaded NMOFs. (B) Time-dependent apoptosis of the spheroid MDA-MB-231 cancer cells: (a) shows untreated cells, (b) and (c) shows cells treated with unloaded (**3**)/(**4**)- and (**3**)/(**5**)-locked NMOFs, respectively, (d) shows cells treated with (**3**)/(**4**)-locked, DOX-loaded, NMOFs, and (e) shows cells treated with (**3**)/(**5**)-locked DOX-loaded NMOFs. Scale bars are 300 μm.

We then examined the cytotoxicity of the (**3**)/(**4**)- and (**3**)/(**5**)-capped DOX-loaded NMOFs towards MDA-MB-321 breast cancer cells and normal MCF-10A epithelial breast cells, Fig. 4. We find that after a time interval of three days *ca.* 35% and 45% cell death of the MDA-MB-321 cells is observed in the presence of the DOX-loaded NMOFs capped with the (**3**)/(**4**) or (**3**)/(**5**) units, respectively, while only ≤5% cell death of the MCF-10A normal cells is detected, as shown in Fig. 4(B), panel I. After a time interval of five days impressive cytotoxicity of the modified NMOFs towards the cancer cells is observed, as shown in Fig. 4(B), panel II. The (**3**)/(**4**)- and (**3**)/(**5**)-capped DOX-loaded NMOFs induce *ca.* 45% and 50% cell death of the MDA-MB-231 cells, while only 15% of MCF-10A normal breast cells were affected, respectively. These preliminary cell experiments reveal that the pH-responsive DNA-modified NMOFs have a superior cytotoxicity towards the cancer cells and that the pH-responsive-AS1411 aptamer conjugate reveals a detectable, enhanced cytotoxicity towards the cancer cells. Presumably, the nano-sized dimensions of the MOF nanoparticles allow the preferential permeation of the nanoparticles into the cancer cells *via* the specific binding of the overexpressed nucleolin receptor, illustrated in Fig. 4(A). The conjugated AS1411 aptamer improves the efficacy of the selective permeation of the drug-loaded nanoparticles by cooperative targeting of the nanoparticles to the cancer cells. The effect of the different DOX-loaded carriers on the viability of the MDA-MB-231 cancer cells was further supported by monitoring the continuous time-dependent cytotoxicity of the NMOFs on spheroid aggregates of MDA-MB-231 cancer cells, Fig. 5(A). In these experiments, the cytotoxicity of the NMOFs is monitored by the time-dependent apoptosis of the aggregates. The NMOFs modified with the (**3**)/(**4**) or (**3**)/(**5**) nucleic acid caps did not show significant toxicity toward the cells, as compared to the MDA-MB-231 cancer cells spheroids that were not treated with the NMOFs (Fig. 5(B), curves (b) and (c) *vs.* (a), respectively). In turn, the DOX-loaded NMOFs capped with (**3**)/(**4**) or (**3**)/(**5**) reveal toxicity towards the cancer cells, shown in curves (d) and (e), respectively. Interestingly, the DOX-loaded NMOFs modified with the i-motif/AS1411 conjugates show a two-fold higher cytotoxicity, as compared to the DOX-loaded NMOFs capped with the (**3**)/(**4**) units, which include only the i-motif-responsive unlock units.

**Fig. 6:**
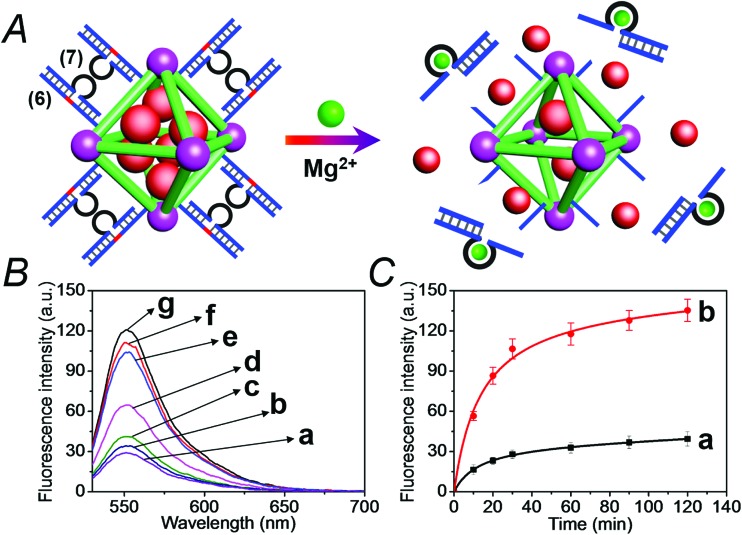
(A) Schematic loading and unloading of the NMOFs with the Rhodamine 6G dye by capping the nanoparticles with the (**6**)/(**7**) duplexes that include the Mg^2+^-ion-dependent loop, and the cleavage of the capping units by Mg^2+^ ions that activate the Mg^2+^-dependent DNAzymes. (B) Fluorescence spectra of the released Rhodamine 6G dye upon treatment of the loaded NMOFs with different concentrations of Mg^2+^ ions for a fixed time interval of 30 minutes: (a) 0 mM, (b) 0.5 mM, (c) 1 mM, (d) 10 mM, (e) 25 mM, (f) 50 mM, and (g) 100 mM. (C) Time-dependent release of the Rhodamine 6G load from the (**6**)/(**7**)-capped dye-loaded NMOFs upon treatment with: (a) 0 mM Mg^2+^ and (b) 25 mM Mg^2+^.

**Fig. 7:**
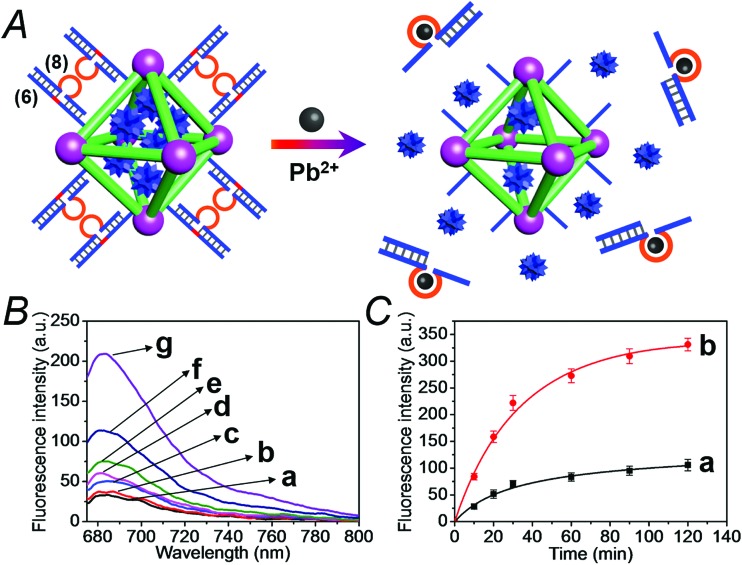
(A) Schematic loading and unloading of the NMOFs with the methylene blue dye by capping the nanoparticles with the (**6**)/(**8**) duplexes that include the Pb^2+^-ion-dependent loop and the cleavage of the capping units by Pb^2+^ ions that activate the Pb^2+^-dependent DNAzymes. (B) Fluorescence spectra of the released methylene blue dye upon treatment of the loaded NMOFs with different concentrations of Pb^2+^ ions for a fixed time interval of 30 minutes: (a) 0 μM, (b) 0.1 μM, (c) 1.0 μM, (d) 10 μM, (e) 50 μM, (f) 100 μM, and (g) 1000 μM. (C) Time-dependent release of the methylene blue loads from the (**6**)/(**8**)-capped dye-loaded NMOFs upon treatment with: (a) 0 μM Pb^2+^ and (b) 100 μM Pb^2+^.

We have further searched for the development of stimuli-responsive DNA-capped, substrate-loaded, NMOFs. Toward this end we applied metal-ion-dependent catalytic nucleic acids as gating units. Metal-ion-dependent catalytic nucleic acids, DNAzymes, have attracted substantial interest in recent years, and different metal ion/nucleic acid conjugates, *e.g.* M^*n*+^ = Mg^2+^, Zn^2+^, Pb^2+^, Hg^2+^, Ni^2+^, and Cu^2+^, have been developed.^11^ Different applications of metal-ion-dependent DNAzymes were reported, including their use as amplifying labels for sensing events,^24^ catalytic unlocking units for the release of drugs from mesoporous SiO_2_ NPs,^25^ for the dissolution of hydrogels and the programmed release of enzymes,^26^ and their use as catalytic optical transducing outputs of logic-gate operations and computing circuits.^27^ Fig. 6(A) depicts the loading of the NMOFs with Rhodamine 6G, their capping with the Mg^2+^-dependent DNAzyme, and the principle of unlocking the NMOFs. The NMOFs were functionalized with the nucleic acid (**6**), using the “click chemistry” method, loaded with Rhodamine 6G, and capped with the Mg^2+^ ion-dependent DNAzyme sequence (**7**). The nucleic acid (**6**) is composed of a nucleobase-modified sequence acting as substrate for the DNAzyme. In the presence of Mg^2+^ ions the DNAzyme is activated, leading to the cleavage of the substrate, unlocking of the NMOFs, and to the release of the dye. Fig. 6(B) shows the fluorescence spectra of the released Rhodamine 6G resulting from the treatment of the NMOFs with variable concentrations of Mg^2+^, for a fixed time of 30 minutes. As the concentration of Mg^2+^ ions increases, the release of the dye is enhanced, and this is consistent with the higher degree of unlocking of the NMOFs by the DNAzyme. Fig. S5† depicts the calibration curve corresponding to the fluorescence of the released dye in the presence of variable concentrations of Mg^2+^ ions (for a fixed time interval of 30 minutes). Fig. 6(C) shows the time-dependent release of the Rhodamine 6G load, in the presence of 25 mM Mg^2+^ ions (curve b), in comparison to the release of the load in the absence of Mg^2+^ ions (curve a). In the presence of Mg^2+^ ions, the release of the load reaches a saturation value after *ca.* 120 minutes. Using an appropriate calibration curve, we estimate that *ca.* 62.7 μmol Rhodamine 6G were released per gram of NMOF nanoparticles. The release of the load in the absence of Mg^2+^ ions reaches a saturation value after *ca.* 120 minutes and no further release is observed after a longer time interval. The low-level release of the dye in the absence of the Mg^2+^ ions is attributed to the release of the load from incomplete (or “defective”) locks associated with surface domains on the NMOFs. The unlocking of the NMOFs is selective towards Mg^2+^ ions, Fig. S6,† and other metal ions do not unlock the NMOFs. A similar concept was applied to unlock methylene blue-loaded NMOFs capped by the Pb^2+^-ion-dependent DNAzyme, shown in Fig. 7(A). In this system, the NMOFs were functionalized *via* the click-chemistry principle, with the ribonucleobase-containing nucleic acid (**6**), acting as substrate for the Pb^2+^-ion-dependent DNAzyme. The (**6**)-modified NMOFs were loaded with methylene blue and capped with the Pb^2+^-ion-dependent sequence (**8**). In the presence of Pb^2+^ ions, the cleavage of the (**6**)/(**8**) duplex capping units proceeds, leading to the unlocking of the NMOFs, and the release of methylene blue. Fig. 7(B) depicts the fluorescence spectra of the methylene blue released, after a fixed time interval of 30 minutes, using variable concentrations of Pb^2+^ ions as unlocking release triggers, and the respective calibration curve is shown in Fig. S7.† As the concentration of Pb^2+^-ions increases, the release process is enhanced, which is consistent with the enhanced unlocking of the capping units. Fig. 7(C) shows the time-dependent release of the methylene blue loads upon subjecting the NMOFs to 100 μM Pb^2+^ (curve b), in comparison to the release of the loads in the absence of the Pb^2+^ ions (curve a). The release profile of methylene blue in the presence of Pb^2+^ ions reveals that after *ca.* 120 minutes the release process reaches a saturation value, implying that the unloading of the dye is completed. Using an appropriate calibration curve, we estimate that *ca.* 61.8 μmol methylene blue were released per gram of nanoparticles within this time interval. As expected, the unlocking of the NMOFs is selective, and other metal ions did not induce the triggered release of the dye from the NMOFs, as shown in Fig. S8.†

**Fig. 8:**
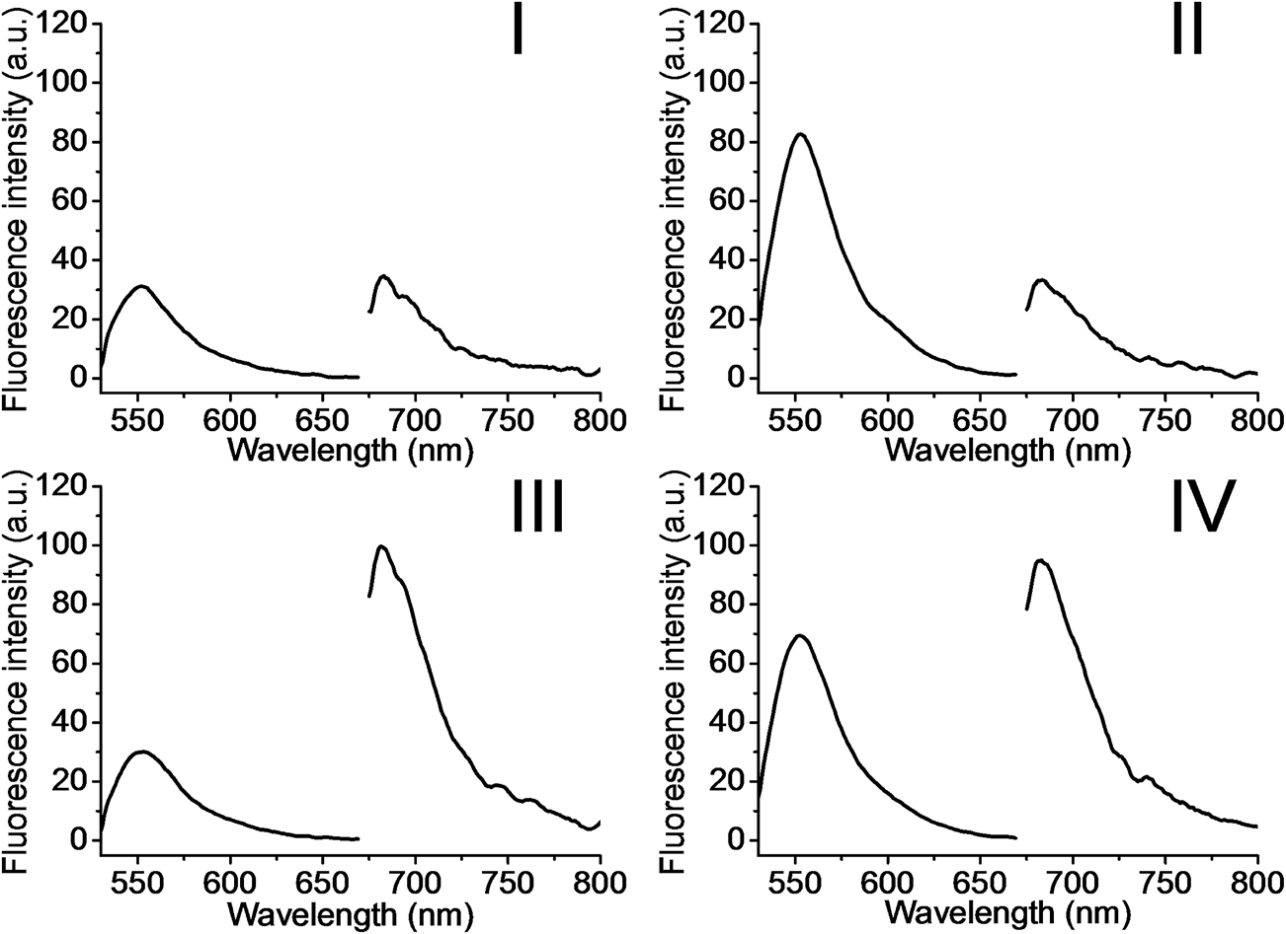
Multiplexed analysis of Mg^2+^ ions and Pb^2+^ ions by the application of the (**6**)/(**7**)- and (**6**)/(**8**)-modified Rhodamine 6G and methylene blue-loaded NMOF mixture. The system is applied also as a logic-gate system, where the Mg^2+^ ions and Pb^2+^ ions act as inputs, and the released loads provide the outputs. The figure depicts the fluorescence output signals of the mixture subjected to the following inputs: panel I: Mg^2+^ ion 0 mM and Pb^2+^ 0 μM; panel II: Mg^2+^ ion 25 mM and Pb^2+^ 0 μM; panel III: Mg^2+^ ion 0 mM and Pb^2+^ 100 μM; panel IV: Mg^2+^ ion 25 mM and Pb^2+^ 100 μM.

The metal-ion-dependent selective release of dye loads from the NMOFs provides a versatile means to detect metal ions, and particularly, a method for the multiplexed analysis of ions using mixtures of NMOFs loaded with different fluorescent labels capped by different metal-ion-dependent DNAzymes. Furthermore, the use of a mixture of NMOFs loaded with two different fluorophores and unlocked by two different metal ions, acting as unlocking triggering units, provides a means for the multiplexed analysis of different ions, where the fluorophore released from the respective NMOF provides the readout signal for the sensing event. Thus, the metal-ion-modified NMOFs may act as modules for logic-gate operations. The two different ion-triggers might act as inputs, and the fluorescence of the released dyes provides the outputs of the logic-gates. Fig. 8 depicts the application of a mixture of the NMOFs loaded with Rhodamine 6G or methylene blue, and capped with the Mg^2+^-ion-dependent DNAzyme and the Pb^2+^-ion-dependent DNAzyme as capping units, respectively, as a model assembly for multiplexed sensing and logic-gate applications. The Mg^2+^ and/or Pb^2+^ ions act as inputs for the system. In the absence of the ions, only the background fluorescence corresponding to the leakage of the fluorophores from the NMOFs is observed (panel I). In the presence of only Mg^2+^ ions or Pb^2+^ ions, the fluorescence of only Rhodamine 6G (*λ*_em_ = 550 nm) or methylene blue (*λ*_em_ = 682 nm) is intensified, shown in panel II and panel III, respectively. In the presence of the inputs, Mg^2+^ and Pb^2+^ ions, the two kinds of NMOFs are unlocked, giving rise to the fluorescence of the two fluorophores, shown in panel IV. Besides demonstrating the ability of the NMOFs to act as functional carriers for multiplexed analysis of ions, the system follows an “AND” logic-gate operation.

**Fig. 9:**
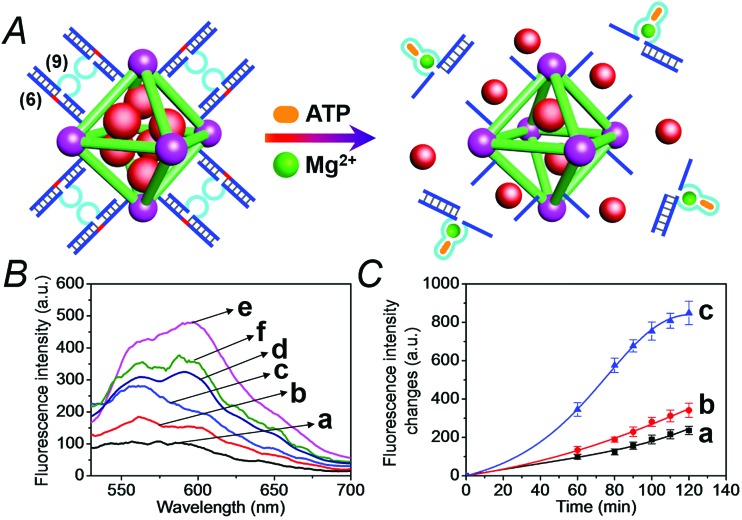
(A) Loading of NMOFs with doxorubicin (DOX) and their capping with DNA scaffolds (**6**)/(**9**) where the strand (**9**) includes the Mg^2+^ ion-dependent loop with an integrated sequence of the ATP aptamer. Unlocking of the capping units *via* the cooperative cleavage of the lock, in the presence of ATP and Mg^2+^ ions. (B) Fluorescence spectra corresponding to the released DOX upon treatment of the (**6**)/(**9**)-capped, DOX-loaded NMOFs with Mg^2+^ and ATP: (a) Mg^2+^ 0 mM and ATP 0 mM; (b) Mg^2+^ 0 mM and ATP 1 mM; (c) Mg^2+^ 1 mM and ATP 1 mM; (d) Mg^2+^ 10 mM and ATP 1 mM; (e) Mg^2+^ 50 mM and ATP 1 mM; (f) Mg^2+^ 2 mM and ATP 3 mM. (C) Time-dependent release of DOX from the (**6**)/(**9**)-capped, DOX-loaded NMOFs in the presence of: (a) Mg^2+^ 0 mM and ATP 0 mM; (b) Mg^2+^ 2 mM and ATP 0 mM; (c) Mg^2+^ 2 mM and ATP 3 mM.

We then applied the DOX-loaded capped NMOFs as functional units for the development of a NMOF carrier responsive to a cancer cell biomarker. In a previous study, we reported on ATP-responsive/Mg^2+^-dependent DNAzyme capped SiO_2_ nanoparticles for the triggered release of the anti-cancer drug doxorubicin.^25^ In this study we made use of the basic elements: (i) ATP is over-expressed in cancer cells due to the enhanced metabolism in these cells, and (ii) for optimal catalytic activities of the Mg^2+^-dependent DNAzyme the precise base-sequence in the loop-region is important. The introduction of a foreign nucleic acid sequence into the loop decreases the activity of the Mg^2+^-dependent DNAzyme, due to the flexibility of the loop-region that reveals a lower affinity toward the Mg^2+^ ions. Nonetheless, it was demonstrated that a foreign sequence corresponding to an aptamer sequence (*e.g.* the anti-ATP aptamer sequence) can, upon formation of the aptamer–ligand complex (*e.g.* the ATP–aptamer complex), act as an auxiliary trigger for the stabilization of the Mg^2+^-ion-dependent DNAzyme loop, and cause re-activation of its high catalytic function. Accordingly, we examined the possibility of designing “smart” ATP-responsive Mg^2+^-ion-dependent DNAzyme-capped DOX-loaded NMOFs for the selective treatment of cancer cells, as shown in Fig. 9(A). In this system, the (**6**)-functionalized NMOFs (where (**6**) consists of the ribonucleobase-modified nucleic acid sequence acting as substrate for the Mg^2+^-dependent DNAzyme) were loaded with doxorubicin (DOX) and capped with the sequence (**9**), which consists of an extended loop composed of the Mg^2+^-dependent DNAzyme subunits, separated by the ATP–aptamer sequence. The resulting (**6**)/(**9**)-capped DOX-loaded NMOFs are expected to reveal inefficient Mg^2+^ ion induced triggered release of the DOX drug, but effective release of the loads in the presence of ATP and Mg^2+^ ions, due to the stabilization of the catalytic loop by the formation of the ATP–aptamer complex. Accordingly, we examined the ATP-triggered release of DOX from the (**6**)/(**9**)-capped NMOFs and compared the release features of the “smart” NMOFs to the (**6**)/(**7**)-capped DOX-loaded NMOFs ((**7**) is the regular Mg^2+^-dependent sequence). Fig. S9† depicts the fluorescence spectra of released DOX upon treatment of the (**6**)/(**7**)-capped DOX-loaded NMOFs with different concentrations of Mg^2+^ ions for a fixed time interval of 60 minutes. As the concentration of Mg^2+^ ions increases, the fluorescence intensity of the released DOX increases, consistent with the enhanced unlocking of the NMOFs. Fig. S10† shows the time-dependent release of DOX upon treatment of the (**6**)/(**7**)-capped NMOFs with 2 mM Mg^2+^ ions, and the release profile is compared to the time-dependent release of DOX from the NMOFs in the absence of added Mg^2+^ ions. Evidently, effective release of DOX proceeds upon treatment of the NMOFs with Mg^2+^ ions. The release of DOX from the NMOFs in the absence of Mg^2+^ ions reaches a low saturation value after *ca.* 100 minutes, and this is attributed to incomplete, (or “defective”) capping sites on the NMOFs. Fig. 9(B) depicts the fluorescence spectra of the released DOX upon the treatment of the (**6**)/(**9**)-capped NMOFs with variable concentrations of Mg^2+^ ions and a fixed concentration of ATP (1 mM) for a fixed time interval of 60 minutes. As the concentration of Mg^2+^ ions increases, the amount of released DOX is higher. Control experiments reveal that in the absence of ATP the unlocking of the (**6**)/(**9**)-capped NMOFs is inefficient in the Mg^2+^-ion concentration range of 1 mM to 50 mM, as shown in Fig. S11.† Furthermore, the unlocking of the (**6**)/(**9**)-capped NMOFs is also controlled by the concentration of ATP, and at a fixed concentration of Mg^2+^ ions (2 mM) the release rate of DOX increases as the concentration of ATP is increased, as shown in Fig. S12.† These results indicate that the unlocking of the (**6**)/(**9**)-capped NMOFs is cooperatively triggered by Mg^2+^ and ATP. While Mg^2+^ ions are essential to induce the release process, the ATP ligand cooperatively enhances the release process. These conclusions are further supported by following the time-dependent release of DOX from the (**6**)/(**9**)-capped NMOFs in the presence and absence of added ATP, shown in Fig. 9(C). While the release of DOX in the presence of 2 mM Mg^2+^ ions is only slightly enhanced as compared to the natural leakage from the NMOFs with no Mg^2+^ ions, the addition of ATP significantly enhances the DOX release, and a 2-fold higher release profile of DOX is observed (for the comparison of the release profiles of the (**6**)/(**7**)- and (**6**)/(**9**)-capped NMOFs see Fig. S13†).

**Fig. 10:**
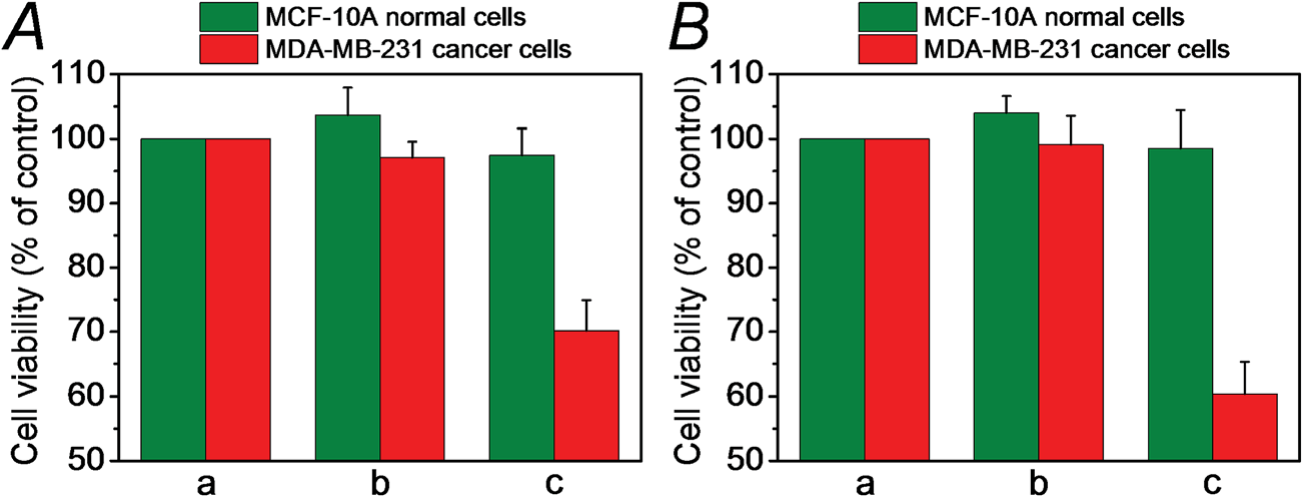
Cytotoxicity of the (**6**)/(**9**)-capped, DOX-loaded NMOFs, and appropriate controls, toward MDA-MB-231 cancer cells (red) and MCF-10A epithelial breast cells (green) upon treatment with: (a) no added NMOFs; (b) addition of empty (**6**)/(**9**)-capped NMOFs; (c) treatment with (**6**)/(**9**)-capped, DOX-loaded NMOFs. (A) After a time interval of three days. (B) After a time interval of five days.

**Fig. 11:**
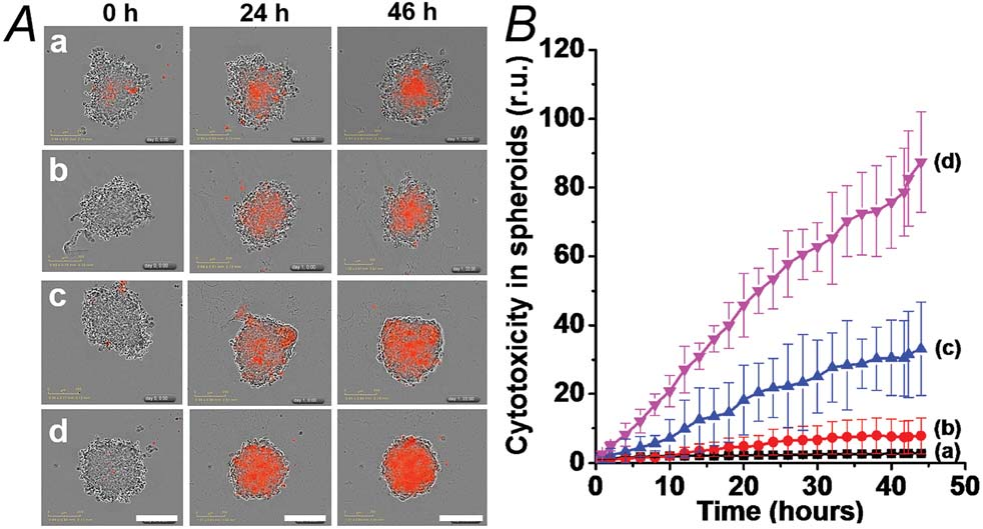
Evaluation of the cytotoxicity of the (**6**)/(**9**)- and (**6**)/(**7**)-capped, DOX-loaded NMOFs, and appropriate controls, by following colorimetrically time-dependent apoptosis of the spheroid MDA-MB-231 aggregates stained with IncuCyte cytotoxicity reagent. (A) Typical apoptosis color images after 0, 24, and 46 hours, corresponding to: (a) untreated cell aggregates; (b) cell aggregates treated with empty (**6**)/(**9**)-capped NMOFs; (c) cell aggregates treated with (**6**)/(**7**)-capped, DOX-loaded NMOFs; (d) cell aggregates treated with (**6**)/(**9**)-capped, DOX-loaded NMOFs. (B) Time-dependent apoptosis of the MDA-MB-231 spheroid aggregates: (a) untreated cells; (b) cell aggregates treated with vacant (**6**)/(**9**)-capped NMOFs; (c) cell aggregates treated with (**6**)/(**7**)-capped, DOX-loaded NMOFs; (d) cell aggregates treated with (**6**)/(**9**)-capped, DOX-loaded NMOFs. Scale bars are 300 μm.

We then examined the cytotoxicity of the (**6**)/(**9**)-capped NMOFs toward MDA-MB-231 breast cancer cells and MCF-10A epithelial normal breast cells, and the results are shown in Fig. 10. While no cytotoxic effect of the DOX-unloaded (**6**)/(**9**)-capped NMOFs on the MDA-MB-231 or MCF-10A was observed after a time interval of five days, the DOX-loaded (**6**)/(**9**)-capped NMOFs had a cytotoxic effect only on the MDA-MB-231 cancer cells. After three days, cell death corresponding to 30% of cells was observed, and this increased to a cell death of 40% after five days. The non-detectable toxicity towards the normal MCF-10A cells is attributed to inefficient permeation of the NMOFs into the normal cells and to the lack of release of DOX by any permeated NMOFs, due to the low concentration of ATP in the cells. The toxicity towards the cancer cells is attributed to the cooperative functions of the over-expressed ATP in cancer cells in the unlocking of the NMOFs and the release of DOX. It should be noted that the (**6**)/(**7**)-capped DOX-loaded NMOFs revealed lower cytotoxicity toward the MDA-MB-231 cancer cells, as compared to that of the (**6**)/(**9**)-capped DOX-loaded NMOFs (15% cell death as compared to 30% cell death, respectively). This difference is attributed to the low concentration of intracellular Mg^2+^ ions, which results in the unlocking process and release of DOX being inefficient. Presumably, the cooperative ATP-driven rigidification of the loop in the (**6**)/(**9**)-capped NMOFs enhances the affinity of intracellular Mg^2+^ ions toward the loop domain, thereby improving the DOX release process. Similar results were obtained upon examining the time-dependent cytotoxicity of the (**6**)/(**7**)- and (**6**)/(**9**)-DOX-loaded NMOFs on aggregated 3D spheroid cancer cells (*cf.* Fig. 11 and accompanying discussion).

The synergistic superior cytotoxic effect of ATP and Mg^2+^ ions on the cytotoxicity of (**6**)/(**9**)-capped NMOFs toward the MDA-MB-231 cells, as compared to the cytotoxicity of (**6**)/(**7**)-capped NMOFs unlocked only by Mg^2+^ ions, were confirmed by following the time-dependent apoptosis of the cell aggregates using the cell IncuCyte system,^28^ as shown in Fig. 11. In this experiment, cell spheroids are treated with the respective NMOFs and the IncuCyte® Red cytotoxicity reagent that probes the apoptosis of cells at the time interval of incubation (see details in Experimental section). Fig. 11(A) depicts the colors of the spheroid cell aggregates treated with different NMOFs for different time intervals (the intensity of the red color reflects the degree of cell death). Evidently, the cancer cells spheroids treated with the DOX-loaded, (**6**)/(**9**)-capped NMOFs reveal substantially high cell death as compared to the spheroids treated with (**6**)/(**7**)-capped NMOFs. Fig. 11(B) shows the time-dependent apoptosis of the MDA-MB-231 cells upon treatment with the different NMOFs. While the DOX-vacant NMOFs capped with (**6**)/(**9**) do not show cytotoxicity (curve (b)), the DOX loaded (**6**)/(**9**)-capped NMOFs show high cytotoxicity towards the cancer cells (curve (d)). The cytotoxicity of the (**6**)/(**9**)-capped NMOFs is superior to the cytotoxicity of the (**6**)/(**7**)-capped NMOFs (curve (d) *vs.* curve (c), respectively).

## Conclusions

In summary, the present study has introduced nucleic acid-functionalized metal–organic framework nanoparticles, NMOFs, as stimuli-responsive carriers of drugs or analoges of drugs. We have developed a versatile methodology to covalently link nucleic acids to the NMOFs, integrate the loads in the NMOFs, and use the nucleic acid tethers as anchoring sites for capping of the NMOFs with stimuli-responsive units that allow the unlocking of the NMOFs and the release of the loads. Two different stimuli-responsive capped NMOFs were introduced. One NMOF carrier included a pH-responsive nucleic acid duplex as a lock. At an acidic pH, the duplex DNA capping units were separated through the reconfiguration of one of the duplex constituents to the i-motif structure, a process that unlocked the capping units and allowed the release of the loads. The second stimuli-responsive NMOF involved the association of a sequence-specific metal-dependent DNAzyme sequence on ribonucleobase modified tethers linked to the NMOFs. In the presence of Mg^2+^ ions or Pb^2+^ ions the catalyzed cleavage of the locks led to the release of the loads. Besides the new fundamental mechanisms to unlock the loaded NMOFs, a method to target the stimuli-responsive NMOFs to the cancer cells was developed *via* the conjugation of the AS1411 aptamer sequence to the stimuli-responsive nucleic acid. The AS1411 aptamer sequence binds to the nucleolin biomarker associated with the cell boundaries of different cancer cells thereby enhancing the permeation of the NMOFs into cancer cells. Furthermore, the design of “smart” Mg^2+^-ion-dependent DNAzyme-capped NMOFs that are unlocked by cooperative triggers (Mg^2+^/ATP) represents a novel approach for a sense-and-treat anti-cancer drug carrier.

The study has highlighted the loading of the different NMOFs with the anti-cancer drug doxorubicin, and the cytotoxicity of the drug-loaded NMOFs toward MDA-MB-231 breast cancer cells and MCF-10A epithelial normal breast cells. Preliminary cell experiments revealed impressive selective cytotoxicity towards the cancer cells. This selective cytotoxicity was attributed to the enhanced permeation of the NMOFs into the cancer cells by the receptor-mediated endocytosis and to the targeted permeation of the NMOFs into the cells. These results suggest that many other drugs, and specifically anti-cancer drugs, could be incorporated into the stimuli-responsive NMOFs. Also, many different NMOFs could act as stimuli-responsive carriers. Beyond the use of the nucleic acid-capped NMOFs as selective drug carriers, the application of the NMOFs for multiplexed sensing and logic-gate operation was demonstrated.

## Experimental section

The sequences of the nucleic acids that were used in the study are:

(**2**) 5′-NH_2_-(CH_2_)_6_-AGTAGGGTTAGG-3′

(**3**) 5′-DBCO-AGTAGGGTTAGG-3′

(**4**) 5′-CCCTAACCCTAACCCTAACCCTACT-3′

(**5**) 5′-GGTGGTGGTGGTTGTGGTGGTGGTGGCCCTAACCCTAACCCTAACCCTACT-3′

(**6**) 5′-NH_2_-(CH_2_)_6_-CAACAACATrAGGACATAGAAGAAGAAG-3′

(**7**) 5′-CTTCTTCTTCTATGTCAGCGATCCGGAACGGCACCCATGTTGTTGTTG-3′

(**8**) 5′-CTTCTTCTTCTATGTCTCCGAGCCGGTCGAATGTTGTTG-3′

(**9**) 5′-CTTCTTCTTCTATGTCAGCGATCCTGGGGGAGTATTGCGGAGGAAGGCACCCATGTTGTTGTTG-3′

The detailed list of materials used in the study, the synthetic protocol to prepare **1**, the synthesis of NMOFs, and the preparation of the N_3_-functionalized NMOFs, are described in the ESI.†

### Loading of the nucleic acid (**3**)-functionalized NMOFs

For the (**3**)-functionalized NMOFs, 5 mg of NMOFs was incubated with methylene blue (0.5 mg mL^–1^) or the anti-cancer drug doxorubicin (1.0 mg mL^–1^) for 24 h in 2 mL of PBS buffer solution (10 mM, pH = 7.4), and this corresponded to the unlocked form of the nucleic acid (**3**)-functionalized NMOFs. Subsequently, the NMOFs were transferred to a buffer solution and hybridized with nucleic acid **4** or **5**, respectively, to yield the locked (**3**)/(**4**) or (**3**)/(**5**) DNA-functionalized NMOFs loaded with the dye or drug. After 12 h, the NMOFs were washed several times to remove the excess and non-specifically bound methylene blue or doxorubicin.

### pH-induced unlocking of the (**3**)/(**4**)- or (**3**)/(**5**)-functionalized NMOFs and the release of the encapsulated loads

The pH-responsive methylene blue-loaded NMOFs (1 mg mL^–1^) were subjected to buffer solutions, at pH = 7.4 or pH = 5.0. At appropriate time intervals, samples of the mixture are centrifuged to precipitate the NMOFs (10 000 rpm for 10 minutes). The fluorescence of the released load in the supernatant solution was measured using a Cary Eclipse Fluorescence Spectrophotometer (Varian Inc.).

### Metal ion-induced unlocking of nucleic acid (**6**)-functionalized NMOFs and the release of the encapsulated loads

The detailed protocol for the loading the (**6**)-functionalized NMOFs with Rhodamine 6G, methylene blue or doxorubicin is provided in the ESI.† The duplex DNA-capped, Rhodamine 6G or methylene blue loaded functionalized NMOFs, at a concentration corresponding to 1 mg mL^–1^, were subjected to the respective ions to unlock the NMOFs and release the loads. The NMOF solutions were treated with different concentrations of Mg^2+^ or Pb^2+^ ions, respectively. At time intervals, the respective sample solutions were centrifuged to precipitate the NMOFs (10 000 rpm for 10 minutes), and the fluorescence of the released loads in the supernatant solutions was measured.

### Parallel operation of the NMOFs using Mg^2+^ ions and Pb^2+^ ions as inputs

The parallel operation of the mixture of (**6**)/(**7**)- and (**6**)/(**8**)-capped NMOFs by unlocking the capping units with Mg^2+^ ions and Pb^2+^ ions as inputs is described in the ESI.† The AND gate operation was examined in a composite solution that included the Mg^2+^-responsive Rhodamine 6G-loaded NMOFs and the Pb^2+^-responsive methylene blue-loaded NMOFs. The appropriate input triggers were Mg^2+^ and/or Pb^2+^ ions: (0, 0), (1, 0), (0, 1), and (1, 1). Digital “1” levels of Mg^2+^ and Pb^2+^ were 25 × 10^–3^ mM and 100 × 10^–6^ μM, respectively. After incubation of the respective samples for 30 minutes, the solutions were centrifuged to precipitate the NMOFs, and the fluorescence of the released loads in the supernatant solution was measured.

### Mg^2+^ and ATP-induced unlocking of the (**6**)/(**9**)-functionalized NMOFs and the release of the encapsulated loads

The (**6**)/(**9**)-capped NMOFs loaded with Rhodamine 6G or DOX (1 mg mL^–1^) were treated with the appropriate concentrations of Mg^2+^ ions and ATP. At defined time intervals, samples of the NMOFs were centrifuged (10 000 rpm) to precipitate the NMOFs, and the fluorescence of the released loads in the supernatant solutions was measured.

A detailed description of the experiments probing the cytotoxicity of the different DOX-loaded NMOFs to the MDA-MB-231 malignant breast cells and MCF-10A epithelial breast cells is described in the ESI.†

### Evaluation the cytotoxicity of cancer cell 3D-spheroids

The NMOF-induced cytotoxicity of 3D-spheroids of the MDA-MB-231 cancer cells was evaluated using the IncuCyte Zoom system (Essen Bioscience).^28^ The MDA-MB-231 cells were seeded in a 96 well ULA plate (Corning 7007) at a density of 2 × 10^3^, and allowed to culture for an additional 3 days to form the spheroids. The resulting spheroids were then treated with the respective NMOFs and the IncuCyte® Red cytotoxicity reagent (Essen Bioscience Cat #4632) to probe the apoptosis of cells. Cell images were recorded every two hours, and the resulting red color, reflecting cell death, was analyzed with the IncuCyte software. This allowed the evaluation of the time-dependent apoptosis of the cells.

## References

[cit1] Wang Z., Cohen S. M. (2009). Chem. Soc. Rev..

[cit2] Zhao M. T., Yuan K., Wang Y., Li G. D., Guo J., Gu L., Hu W. P., Zhao H. J., Tang Z. Y. (2016). Nature.

[cit3] Yang S. J., Kim T., Im J. H., Kim Y. S., Lee K., Jung H., Park C. R. (2012). Chem. Mater..

[cit4] He C., Lu K., Liu D., Lin W. (2014). J. Am. Chem. Soc..

[cit5] Wu L. L., Wang Z., Zhao S. N., Meng X., Song X. Z., Feng J., Song S. Y., Zhang H. J. (2016). Chem.–Eur. J..

[cit6] Hurd J. A., Vaidhyanathan R., Thangadurai V., Ratcliffe C. I., Moudrakovski I. L., Shimizu G. K. H. (2009). Nat. Chem..

[cit7] Wang F., Lu C. H., Willner I. (2014). Chem. Rev..

[cit8] Leroy J. L., Guéron M., Mergny J. L., Hélène C. (1994). Nucleic Acids Res..

[cit9] Li T., Wang E., Dong S. (2010). Anal. Chem..

[cit10] Li T., Wang E., Dong S. (2009). J. Am. Chem. Soc..

[cit11] Liu J., Cao Z., Lu Y. (2009). Chem. Rev..

[cit12] Lu C. H., Willner I. (2015). Angew. Chem., Int. Ed..

[cit13] Lu C. H., Qi X. J., Orbach R., Yang H. H., Mironi-Harpaz I., Seliktar D., Willner I. (2013). Nano Lett..

[cit14] Liao W. C., Lu C. H., Hartmann R., Wang F. A., Sohn Y. S., Parak W. J., Willner I. (2015). ACS Nano.

[cit15] Rodríguez-Pulido A., Kondrachuk A., Prusty D. K., Gao J., Loi M. A., Herrmann A. (2013). Angew. Chem., Int. Ed..

[cit16] Chen L., Di J., Cao C., Zhao Y., Ma Y., Luo J., Wen Y., Song W., Song Y., Jiang L. (2011). Chem. Commun..

[cit17] Zhang Z., Balogh D., Wang F., Sung S. Y., Nechushtai R., Willner I. (2013). ACS Nano.

[cit18] Liao W. C., Sohn Y. S., Riutin M., Cecconello A., Parak W. J., Nechushtai R., Willner I. (2016). Adv. Funct. Mater..

[cit19] Zhang Z., Wang F., Balogh D., Willner I. (2014). J. Mater. Chem. B.

[cit20] Meng X., Gui B., Yuan D., Zeller M., Wang C. (2016). Sci. Adv..

[cit21] Kahn J. S., Freage L., Enkin N., Garcia M. A., Willner I. (2017). Adv. Mater..

[cit22] He C., Lu K., Lin W. (2014). J. Am. Chem. Soc..

[cit23] Yang X., Liu X., Liu Z., Pu F., Ren J., Qu X. (2012). Adv. Mater..

[cit24] Wang F., Elbaz J., Orbach R., Magen N., Willner I. (2011). J. Am. Chem. Soc..

[cit25] Zhang Z., Balogh D., Wang F., Willner I. (2013). J. Am. Chem. Soc..

[cit26] Lilienthal S., Shpilt Z., Wang F., Orbach R., Willner I. (2015). ACS Appl. Mater. Interfaces.

[cit27] Elbaz J., Lioubashevski O., Wang F., Remacle F., Levine R. D., Willner I. (2010). Nat. Nanotechnol..

[cit28] Gautam P., Karhinen L., Szwajda A., Jha S. K., Yadav B., Aittokallio T., Wennerberg K. (2016). Mol. Cancer.

