# Supplementary material for: Stimuli-responsive nucleic acid-functionalized metal–organic framework nanoparticles using pH- and metal-ion-dependent DNAzymes as locks
Source: Chem Sci. 2017 Jun 6;8(8):5769–80. doi: 10.1039/c7sc01765k (PMC5621505; doi:10.1039/c7sc01765k)
Supplement: Supplementary file 1 [file SC-008-C7SC01765K-s001.pdf]

Electronic Supplementary Material (ESI) for Chemical Science.
This journal is © The Royal Society of Chemistry 2017

Electronic Supplementary Information

of

Stimuli-Responsive Nucleic Acid-Functionalized Metal-Organic-Framework Nanoparticles Using pH- and Metal-Ion Dependent DNAzymes as Locks

Wei-Hai Chen,[1†] Xu Yu,[1†] Alessandro Cecconello,[1] Yang Sung Sohn,[2] Rachel Nechushtai,[2] and Itamar Willner[1,*]

[1]Institute of Chemistry, Center for Nanoscience and Nanotechnology, The Hebrew University of Jerusalem, Jerusalem 91904, Israel

[2]Institute of Life Science, The Hebrew University of Jerusalem, Jerusalem 91904, Israel

*To whom correspondence should be addressed. Tel: 972-2-6585272. Fax: 972-2-6527715.

E-mail: willnea@vms.huji.ac.il

†These authors contributed equally to this work.

Experimental Section

Materials: Rhodamine 6G, Methylene Blue, adenosine-5'-triphosphate (ATP), doxorubicin hydrochloride, magnesium (II) chloride, zinc (II) acetate dihydrate, lead (II) acetate trihydrate, calcium (II) acetate hydrate, strontium (II) chloride hexahydrate, nickel (II) acetate tetrahydrate, mercury(II) acetate, 4-(methoxycarbonyl)-phenylboronic acid, 2,5-dibromoaniline, cesium fluoride (CsF), anhydrous tetrahydrofuran (THF), dimethylformamide (DMF), trifluoroacetic acid, palladium(II) acetate ($Pd(OAc)_2$), triphenylphosphine (PPh_3), dibenzocyclooctyne-sulfo-N-hydroxysuccinimidyl ester (DBCO-sulfo-NHS), tert-butyl nitrite (tBuONO), azidotrimethylsilane ($TMSN_3$) were purchased from Aldrich/Sigma. Ultrapure water was obtained by a NANOpure Diamond instrument (Barnstead International, Dubuque, IA, USA). All oligonucleotides were synthesized, standard desalting purified, and freeze-dried by Integrated DNA Technologies, Inc.

Synthesis of amine-functionalized triphenyl carboxylic acid (1): Amino-functionalized triphenyl carboxylic acid was synthesized according to previously reported method with minor modification.[1] 2,5-Dibromoaniline (1.00 g, 4.0 mmol), 4-(methoxycarbonyl)-phenylboronic acid (2.2 g, 12.25 mmol) and CsF (2.91 g, 19 mmol) were dissolved in 50 mL of anhydrous THF under nitrogen. Then $Pd(OAc)_2$ (0.30 g, 1.35 mmol) and PPh_3 (0.8 g, 3.05 mmol) were added to the mixture that was heated at 50 °C for 48 h. The product was purified by water/dichloromethane extraction and silica gel column chromatography (dichloromethane: ethyl ether = 50: 1 with 0.25% triethylamine). The resulting amino-triphenyldicarboxyl methyl ester (1.2 g, 3.32 mmol) was suspended in 150 mL of THF and heated to 40 °C. Then 80 mL of 5.5 M

KOH in methanol solution were added to the mixture that was stirred at 40 °C for overnight. The resulting white solid was collected by centrifugation, and then treated with 10 mL of trifluoroacetic acid in 80 mL of THF at room temperature for 2 h. The yellow solid product (amino-functionalized triphenyl dicarboxylic acid) was filtered by vacuum filtration and washed with THF, methanol and ether, respectively. ^{1}H NMR (DMSO-d_6): δ=12.99 (br, 2H), 8.03 (m, 4H), 7.78 (d, 2H), 7.62 (d, 2H), 7.17 (d, 2H), 7.01 (dd, 1H), 5.12 (br, 2H).

Synthesis of NMOFs: The NMOFs were synthesized according to the previously reported method.[1] For the preparation of the NMOFs, 42 mg of amine-functionalized triphenyl carboxylic acid (**1**) and 60 mg of $ZrCl_4$ were mixed in 100 mL of DMF. 5 mL of CH_3COOH were added to the mixture that was kept in an 80 °C oven for 5 days. The resulting NMOFs were collected by centrifugation and washed with DMF, triethylamine/ethanol (1:20, V/V), and ethanol, respectively.

Synthesis of NMOFs with azide group (NMOF-N_3): The dried NMOFs (20 mg) were placed in a vial that included 5 mL of THF. Then, 1.5 mL of the tert-butyl nitrite (tBuONO) and 1.4 mL of the azidotrimethylsilane ($TMSN_3$) were added. The mixture was incubated at the room temperature overnight to produce the azide intermediate corresponding compound (NMOF-N_3).

Synthesis of DBCO-functionalized nucleic acid (2) or (6): The covalent tethering of DBCO to nucleic acid (**2**) or (**6**), was achieved by reacting 40 μL of 1 mM nucleic acid (**2**) or (**6**) with 100 μL of 4 mM DBCO-sulfo-NHS in PBS buffer by shaking the

mixture overnight. The solution is filtered with MicroSpin G-25 columns (GE) to separate out and obtain the pure DBCO-DNA.

Synthesis of nucleic acid (3) or (6)-functionalized NMOFs: NMOF-N_3 nanoparticles (10 mg, 1 mL) were added to an aqueous solution of DBCO-modified nucleic acid (**1**) or (**5**) (100 nmol, 0.5 mL), respectively. The resulting solutions were stirred at 40 °C for 72 h. NaCl was added to the solution over 6 h in three equal aliquots to a final concentration of 0.5 M. The nucleic acid (**3**) or (**6**)-functionalized NMOFs were washed three times with a PBS buffer to remove unbound DNA. The UV absorbance of the wash at 260 nm was measured to determine the amount of DNA coating the NMOFs, using the UV-2450 UV-VIS Spectrophotometer (Shimadzu).

Loading of the nucleic acid (6)-functionalized NMOFs: The (**6**)-functionalized NMOFs, 5 mg in 2 mL PBS buffer solution (pH = 7.4), were incubated with Rhodamine 6G (0.5 mg/mL), Methylene Blue (0.5 mg/mL) or the anticancer drug doxorubicin (1.0 mg/mL) for 24 h. The NMOFs were then transferred to a buffer solution and hybridized with nucleic acid (**7**), (**8**) or (**9**), respectively, leading to the locked state of the loaded duplex DNA-functionalized NMOFs. After 12 h, the NMOFs were washed several times to remove the excess and nonspecifically bound Rhodamine 6G, Methylene Blue or doxorubicin.

Evaluation of the loading of Methylene Blue in the NMOFs: Fig. S2 shows the calibration curve corresponding to the fluorescence intensities as a function of concentration of Methylene Blue at pH = 5.0. The saturated fluorescence of the released Methylene Blue from 10 μg of NMOFs (dilutes 30 times before a measurement) at pH

= 5.0, reveals a fluorescence intensity of ca. 500 a.u, Fig. S1(A). Placing this value in the calibration curve shows in Fig. S2 (marked with arrow) indicates the release of 2.48 μM that translates to a loading of 72.2 μmole Methylene Blue·gram^{-1} of NMOFs.

Evaluation of the loading of DOX in the NMOFs: Fig. S3 shows the calibration curve corresponding to the fluorescence intensities as a function of concentration of DOX at pH = 5.0. The saturated fluorescence of the released DOX from 10 μg of NMOFs (dilutes 30 times before a measurement) at pH = 5.0, reveals a fluorescence intensity of ca. 1100 a.u, Fig. 2(C). Placing this value in the calibration curve shows in Fig. S3 (marked with arrow) indicates the release of 1.76 μM that translates to a loading of 52.8 μmole DOX·gram^{-1} of NMOFs.

Cell culture: Malignant breast epithelial cells (MDA-MB-231) were grown in 5% CO_2 RPMI medium 1640 supplemented with 10% FCS, L-glutamine, and antibiotics (Biological Industries). Normal breast cells (MCF-10A) were maintained in complete growth medium consisting of 1:1 mixture of Dulbecco's modified Eagle's medium and Ham's F12 medium supplemented with horse serum (5%), epidermal growth factor (20 ng/mL), cholera toxin (CT, 0.1 μg/mg), insulin (10 μg/mL), hydrocortisone (500 ng/mL), and penicillin/streptomycin (1 unit/mL). Cells were plated one day prior to the experiment on 24-well plates or on microscopic slides glued to perforated 3 cm diameter tissue culture plates for cell viability or microscopic measurement.

Cell uptake of NMOFs: Normal breast cells (MCF-10A) and malignant breast epithelial cells (MDA-MB-231) were planted at a density of 8 × 10^5 cells in glass-bottomed petri-dish (9.5 cm^2 growth area) with 2 mL medium and incubated with the

DOX-loaded NMOFs (10 μg/mL) in the growth medium for 6 h. Subsequently, the cells were washed with DMEM-HEPES, and the nuclei were stained with DAPI (4,6-diamidino-2-phenylindole). The fluorescence of DOX was measured by epi-fluorescence microscopy (Nikon TE2000 microscope) equipped with opti-grid. Image analysis was performed using Image-J and Volocity programs.

Cell viability experiments: Cell viability was assayed after incubation of the DOX-loaded NMOFs with MCF-10A or MDA-MB-231 cells planted at a density of 1.8×10^5 cells per well, in 24-well plates (1.9 cm^2 growth area). After seeding of the cells for 12 hours, the cells were incubated with the DOX-loaded NMOFs for 6 h, followed by intensive washing. The cells were then incubated for 3 days or 5 days with the growth medium and the cell viability was determined with the fluorescent redox probe, Alamar Blue. The fluorescence of Alamar Blue was recorded on a plate-reader (Tecan Safire) after 1 h of incubation at 37 °C (λex = 560 nm; λem = 590 nm).

Reference

1. C. He, K. Lu, D. Liu, W. Lin, *J. Am. Chem. Soc.* **2014**, *136*, 5181.

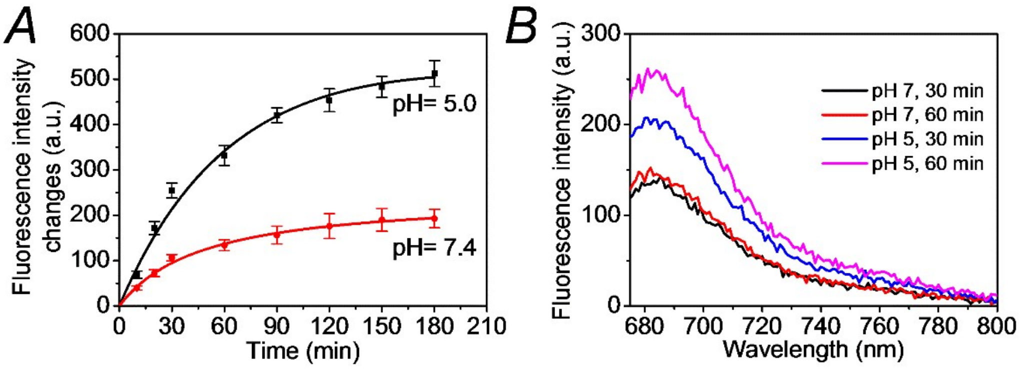

Fig. S1 (A) Time-dependent fluorescence upon treatment of the Methylene Blue-loaded, (**3**)/(**4**)-capped NMOFs at pH = 7.4 and pH = 5.0. The fluorescence corresponds to the released Methylene Blue. (B) Fluorescence spectra of the released Methylene Blue from the (**3**)/(**4**)-capped NMOFs.

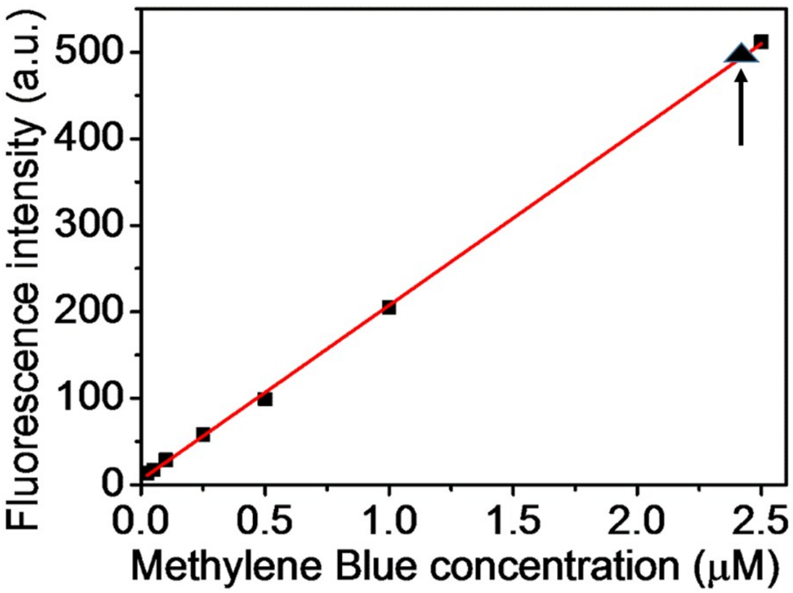

Fig. S2 The calibration curve corresponding to the fluorescence intensities as a function of concentration of Methylene Blue. The arrow indicates the saturated fluorescence of the released Methylene Blue from NMOFs, at pH = 5.0.

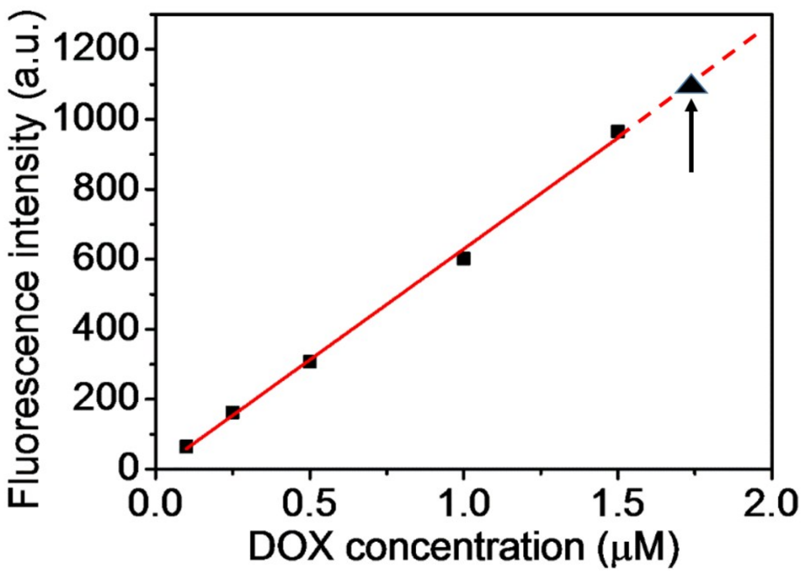

Fig. S3 The calibration curve corresponding to the fluorescence intensities as a function of the concentration of DOX. The arrow indicates the saturated fluorescence of the released DOX from NMOFs, at pH = 5.0.

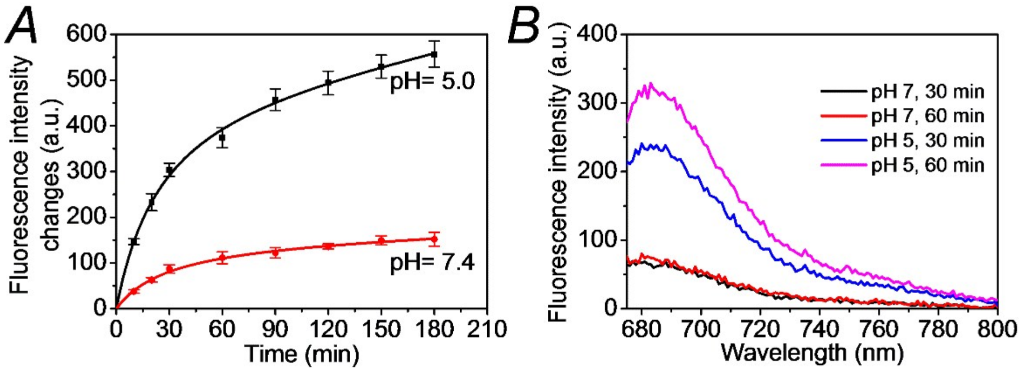

Fig. S4 (A) Time-dependent fluorescence upon treatment of the Methylene Blue-loaded, (**3**)/(**5**)-capped NMOFs at pH = 7.4 and pH = 5.0. The fluorescence corresponds to the released Methylene Blue. (B) Fluorescence spectra of the released Methylene Blue from the (**3**)/(**5**)-capped NMOFs.

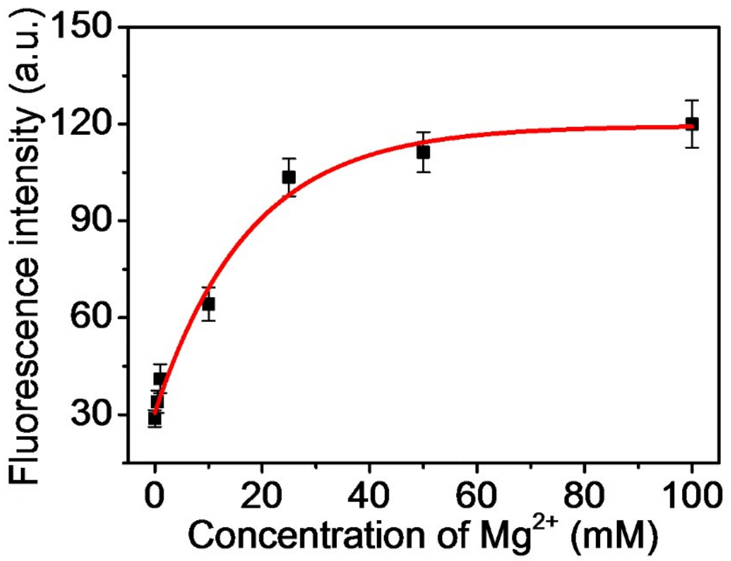

Fig. S5 The calibration curve corresponding to the fluorescence of the released dye from Rhodamine 6G-loaded, (**6**)/(**7**)-capped NMOFs in the presence of variable concentration of Mg^{2+} ions.

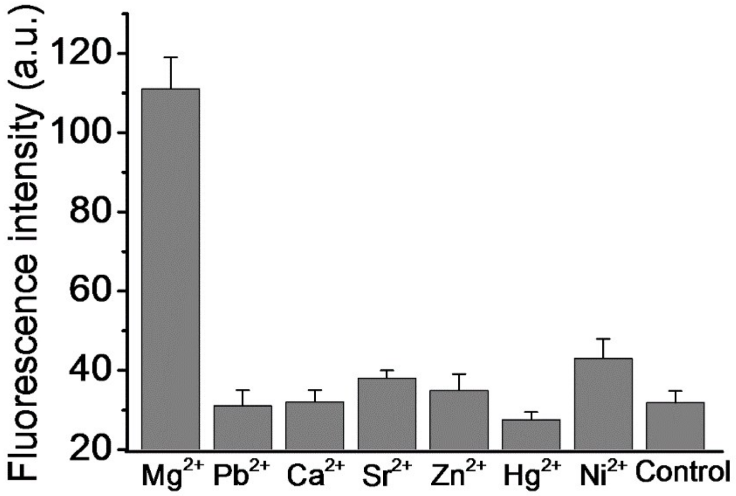

Fig. S6 Fluorescence changes of Rhodamine 6G upon the treatment of the (**6**)/(**7**)-functionalized NMOFs with different metal ions (25 mM) for a time-interval of 60 minutes.

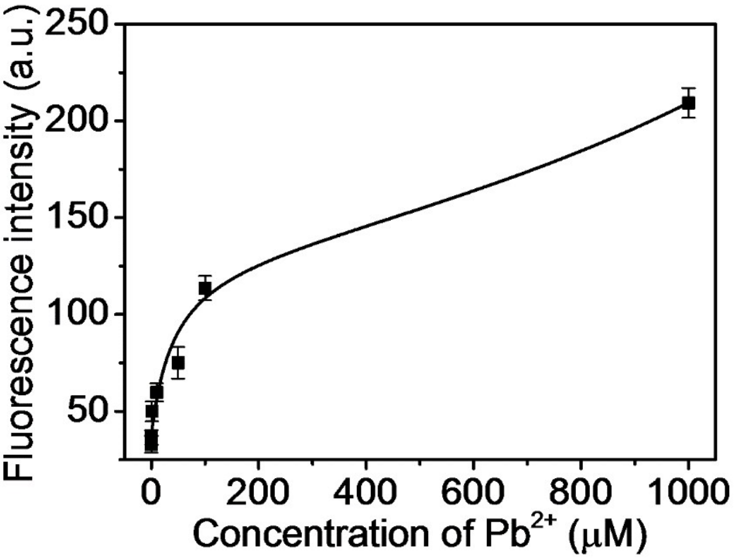

Fig. S7 The calibration curve corresponding to the fluorescence of the released dye from Methylene Blue-loaded, (**6**)/(**8**)-capped NMOFs in the presence of variable concentration of Pb^{2+} ions.

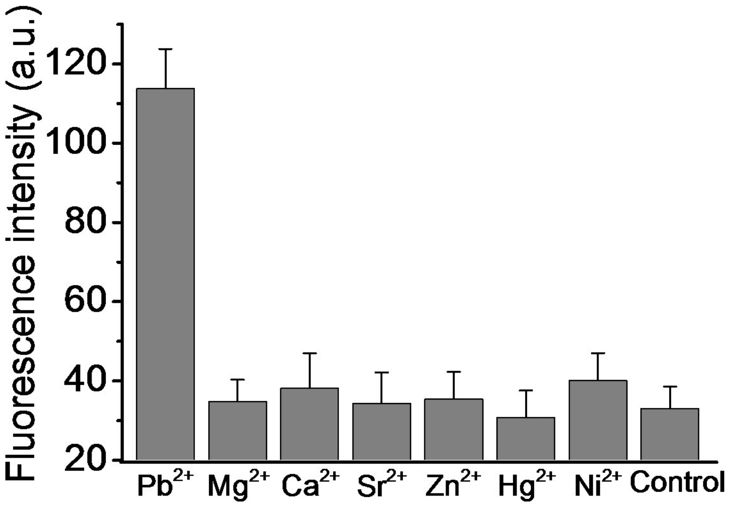

Fig. S8 Fluorescence changes of Methylene Blue upon the treatment of the (**6**)/(**8**)-functionalized NMOFs with different metal ions (100 μM) for a time-interval of 60 minutes.

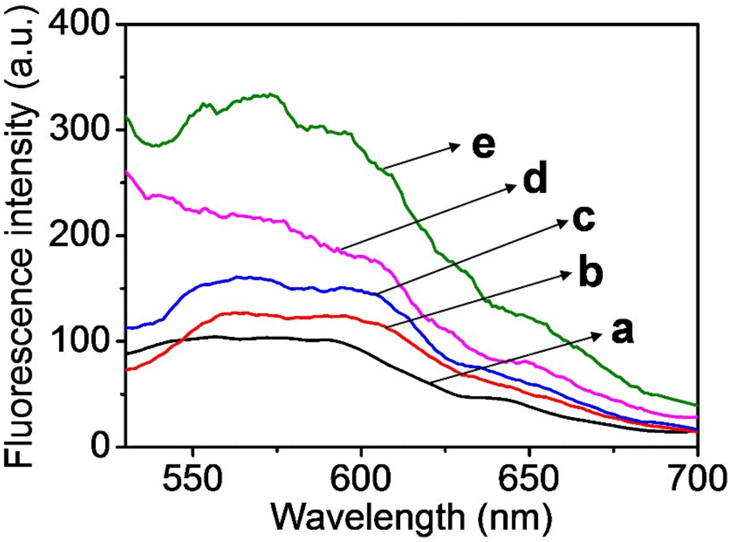

Fig. S9 The fluorescence spectra of released DOX upon treatment of the (**6**)/(**7**) capped DOX-loaded NMOFs with different concentrations of Mg^{2+}-ions for a fixed time-interval of 60 minutes: (a) 0 mM, (b) 1 mM, (c) 2 mM, (d) 10 mM, (e) 50 mM.

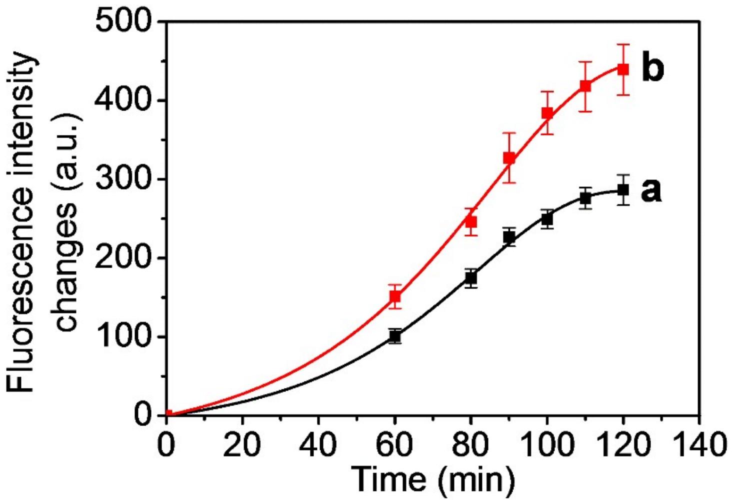

Fig. S10 Time-dependent release of the DOX load from the (**6**)/(**7**)-capped DOX e loaded NMOFs upon treatment with: (a) 0 mM Mg^{2+}, (b) 2 mM Mg^{2+}.

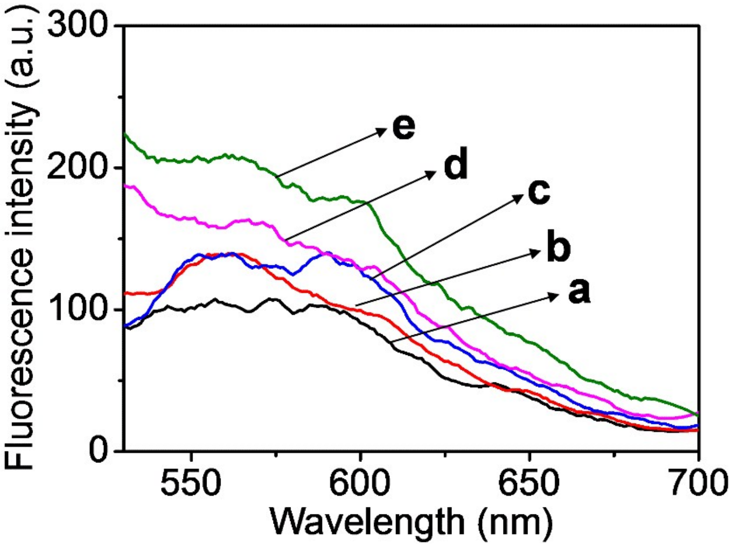

Fig. S11 The fluorescence spectra of released DOX upon treatment of the (**6**)/(**9**) capped DOX loaded NMOFs with different concentrations of Mg^{2+}-ions for a fixed time-interval of 60 minutes: (a) 0 mM, (b) 1 mM, (c) 2 mM, (d) 10 mM, (e) 50 mM.

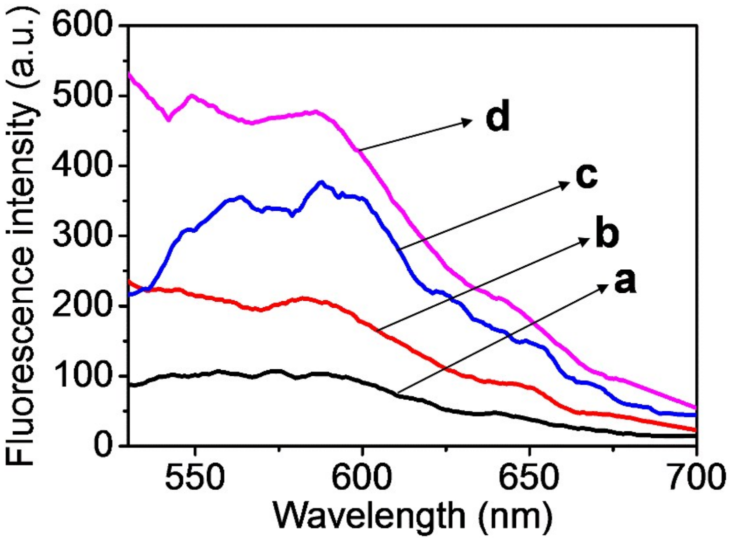

Fig. S12 The fluorescence spectra of released DOX upon treatment of the (**6**)/(**9**) capped DOX loaded NMOFs with different concentrations of ATP/Mg^{2+} for a fixed time-interval of 60 minutes: (a) ATP 0 mM, Mg^{2+} 0 mM, (b) ATP 1 mM, Mg^{2+} 2 mM, (c) ATP 3 mM, Mg^{2+} 2 mM, (d) ATP 10 mM, Mg^{2+} 2 mM.

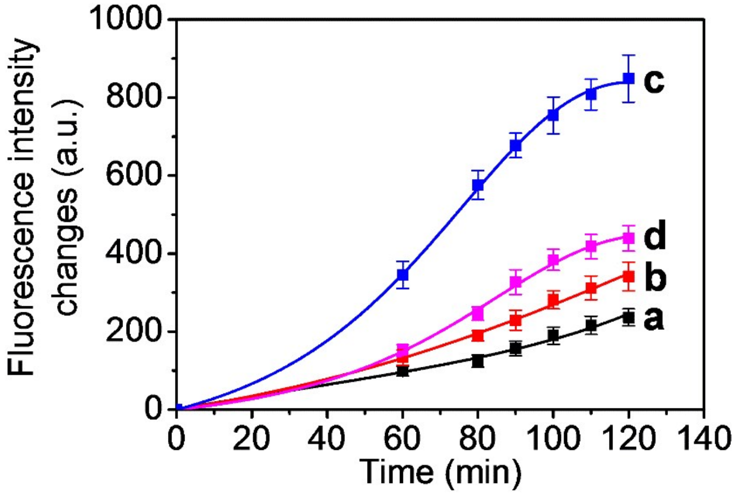

Fig. S13 Time-dependent release of DOX from the (**6**)/(**9**)-capped, DOX-loaded NMOFs in the presence of: (a) Mg^{2+} 0 mM, ATP 0 mM; (b) Mg^{2+} 2 mM, ATP 0 mM; (c) Mg^{2+} 2 mM, ATP 3 mM, (d) Time-dependent release of DOX from the (**6**)/(**7**)-capped, DOX-loaded NMOFs in the presence of Mg^{2+} 2 mM.
